# Cytotoxic B Cells in Relapsing-Remitting Multiple Sclerosis Patients

**DOI:** 10.3389/fimmu.2022.750660

**Published:** 2022-02-07

**Authors:** Vinícius O. Boldrini, Ana M. Marques, Raphael P. S. Quintiliano, Adriel S. Moraes, Carla R. A. V. Stella, Ana Leda F. Longhini, Irene Santos, Marília Andrade, Breno Ferrari, Alfredo Damasceno, Rafael P. D. Carneiro, Carlos Otávio Brandão, Alessandro S. Farias, Leonilda M. B. Santos

**Affiliations:** ^1^ Autoimmune Research Laboratory, Department of Genetics, Evolution, Microbiology and Immunology, Institute of Biology, University of Campinas, Campinas, Brazil; ^2^ Neuroimmunology Unit, Department of Genetics, Evolution, Microbiology and Immunology, Institute of Biology, University of Campinas, Campinas, Brazil; ^3^ Department of Neurology, University of Campinas, Campinas, Brazil; ^4^ Department of Immunology and Rheumatology, University of Alabama at Birmingham, Birmingham, AL, United States; ^5^ MS Clinic of Santa Casa de São Paulo (CATEM), Irmandade da Santa Casa de Misericordia de São Paulo, São Paulo, Brazil; ^6^ National Institute of Science and Technology on Neuroimmunomodulation (INCT-NIM), Rio de Janeiro, Brazil; ^7^ Experimental Medicine Research Cluster (EMRC), São Paulo, Brazil

**Keywords:** cytotoxicity, granzyme B, neuroinflammation, neurodegeneration, MS treatment

## Abstract

**Background:**

Emerging evidence of antibody-independent functions, as well as the clinical efficacy of anti-CD20 depleting therapies, helped to reassess the contribution of B cells during multiple sclerosis (MS) pathogenesis.

**Objective:**

To investigate whether CD19^+^ B cells may share expression of the serine-protease granzyme-B (GzmB), resembling classical cytotoxic CD8^+^ T lymphocytes, in the peripheral blood from relapsing-remitting MS (RRMS) patients.

**Methods:**

In this study, 104 RRMS patients during different treatments and 58 healthy donors were included. CD8, CD19, Runx3, and GzmB expression was assessed by flow cytometry analyses.

**Results:**

RRMS patients during fingolimod (FTY) and natalizumab (NTZ) treatment showed increased percentage of circulating CD8^+^GzmB^+^ T lymphocytes when compared to healthy volunteers. An increase in circulating CD19^+^GzmB^+^ B cells was observed in RRMS patients during FTY and NTZ therapies when compared to glatiramer (GA), untreated RRMS patients, and healthy donors but not when compared to interferon-β (IFN). Moreover, regarding Runx3, the transcriptional factor classically associated with cytotoxicity in CD8^+^ T lymphocytes, the expression of GzmB was significantly higher in CD19^+^Runx3^+^-expressing B cells when compared to CD19^+^Runx3^-^ counterparts in RRMS patients.

**Conclusions:**

CD19^+^ B cells may exhibit cytotoxic behavior resembling CD8^+^ T lymphocytes in MS patients during different treatments. In the future, monitoring “cytotoxic” subsets might become an accessible marker for investigating MS pathophysiology and even for the development of new therapeutic interventions.

## Introduction

Multiple sclerosis (MS) is an autoimmune-mediated demyelinating disease of the central nervous system (CNS). Early evidence showed the presence of CD8^+^ T lymphocytes in the cerebrospinal fluid (CSF) and in perivascular leukocyte infiltration from white matter in chronic and active MS lesions (1–5). Thus, since there are few natural killer (NK) cells compared with T cells in the CSF of MS patients (6) and also effector T populations may be even more potent than NK cells in releasing cytotoxic granules (7); the expression of cytotoxic-associated molecules such as serine-protease granzyme-B (GzmB), during MS, seems to almost be exclusively originating from CD8^+^ T lymphocytes. Interestingly, Runx3, which is a crucial transcriptional factor related to the expression of cytotoxic molecules in effector CD8^+^ T subsets (8, 9), is reported as an MS-associated gene (10). In parallel, neurons express the mannose-6-phosphate receptor (M6PR), responsible for internalizing GzmB, which then makes them vulnerable to cell death triggered by this protease. *In vitro* evidence suggests that serine-protease inhibitors can dampen neuronal cell death associated with GzmB internalization (11). Supporting these findings, it was shown that MS patients exhibit higher GzmB levels in the CSF during relapses that tend to persist higher at 1–3 months into clinical remission (12). Also, increased circulating T lymphocytes with the ability to express GzmB were found in the peripheral blood from relapsing-remitting MS (RRMS) patients treated with fingolimod (FTY), and particularly during relapses, when compared to RRMS patients without FTY (13). Similarly, massive infiltration of cytotoxic CD8^+^GzmB^+^ T lymphocytes was found in the CNS parenchyma from two MS patients who suffered fulminant relapses after natalizumab (NTZ) discontinuation (14, 15). On the other hand, regarding progressive MS courses, not only the CSF from secondary progressive MS (SPMS) patients showed *in vitro* neurotoxicity due to the expression of GzmB (16) but also cytotoxic CD8^+^CD57^+^ T lymphocytes seem to be present in inflamed meninges in these patients with rapidly progressive disease (4). Altogether, these findings reinforce that cytotoxic mechanisms derived from CD8^+^ T lymphocytes are pivotal drivers of CNS damage during MS (12, 17, 18).

Despite this, successful outcomes in the last few years by the use of anti-CD20 monoclonal antibodies (mAbs) (rituximab, ocrelizumab, or ofatumumab) reassessed the importance of B cells during both relapsing-remitting (RRMS) and progressive MS courses (4). Indeed, oligoclonal band (OCB) synthesis, compartmentalized clonal expansion, and increased levels of chemoattractants for B cells and/or plasma cells in the CSF (19–22) were extensively described in MS patients. Nevertheless, since the CD20 molecule is not expressed on pro-B cells or differentiated plasma cells, the beneficial effect of anti-CD20 treatment appears to be extended beyond autoantibody production and release. For instance, in the last few years, increasing evidence supports that B subsets can express and release anti- and pro-inflammatory cytokines, evidencing their antibody-independent functions during MS pathophysiology (23, 24). Considering it, in the present study, we evaluated whether CD19^+^ B subsets may also exhibit the capacity to express and release GzmB similarly resembling the cytotoxic activity described for T lymphocytes in RRMS patients.

## Methods

### Study Participants

A total of 104 RRMS patients [19 Untreated, 15 Glatiramer Acetate (GA), 24 Interferon-β (IFN), 14 FTY, and 32 NTZ], according to the McDonald criteria were recruited in the Neurology Clinic at the University of Campinas Hospital (UNICAMP). Also, 58 healthy subjects were included in the control group (**Table 1**). All subjects signed a term of consent approved by the University Committee for Ethical Research (CAAE: 53022516.3.0000.5404).

**Table 1:** Demographic and baseline clinical characteristics of MS patients and controls.

Subjects	Sample size	Gender ♀:♂	Age	Time after first relapse (years)	Time after last relapse (months)	Treatment duration (years)	EDSS	OCB CSF (+/-)*
Healthy	58	40:18	28 (19-50)	–	–	–	–	
RRMS	104	80:24	37 (18-65)	9 (0.5-32)	27 (0-166)	3.0	2.0 +- 1.9	60/30
*RRMS patients*								
Untreated	19	14:5	27 (18-59)	5 (0.5-19)	4.5 (0-146)	–	1.5 +- 2.0	12/6*
Glatiramer Acetate (GA)	15	13:2	42 (23-58)	12.5 (1-32)	21 (5- 93)	4.0	1.5 +- 1.4	7/5*
Interferon-β (IFN)	24	20:4	41 (28-65)	12.5 (1-22)	40 (1-166)	6.5	2.0 +- 1.6	12/8*
Fingolimod (FTY)	14	10:4	39 (22-65)	11 (4-25)	102 (32-132)	3.0	2.0 +- 1.6	8/5*
Natalizumab (NTZ)	32	23:9	35 (23-62)	9 (2-15)	48 (24-120)	2.0	2.0 +- 2.0	21/6*

All data are represented in median (max – min values).

*Not all patients were tested for oligoclonal bands (Tested: n = 90; 66% OCB positive in the CSF).

CSF, Cerebrospinal Fluid; OCBs, Oligoclonal Bands; EDSS, Expanded Disease Scale Status.

### Blood Sample Collection and Lymphocyte Separation

Peripheral blood (25 ml) samples were collected from RRMS patients and healthy volunteers. Peripheral blood mononuclear cells (PBMCs) were separated by Ficoll-Hypaque^®^ gradient and resuspended after centrifugation on RPMI-1640 supplemented with 10% heat-inactivated fetal bovine serum, 100 U/ml penicillin, and 100 μg/ml streptomycin. Then, PBMCs were used fresh or cryopreserved according to each experiment.

### Flow Cytometry Analyses (FCA)

According to each experiment, PBMCs were stained with different anti-human mAbs: CD3-5.5 PerCP (clone SP34-2), CD3 BUV496 (clone UCHT1), CD8 PE (clone RPA-T8), CD8 BUV563 (clone RPAT8), CD19 FITC (clone HIB19), CD19 BV510 (clone SJ25C1), CD20 BV750 (clone 2H7), CD25 BUV805 (clone 2A3), CD27 BV711 (clone M7271), CD28 BUV737 (clone CD28.2), CD38 BB790 (clone HIT2), IgD BUV615 (clone IA6-2), CD45RA BB515 (clone HI100), CD56 APC-R700 (clone NCAM16.2), CD57 PECF594 (clone NK-1), CD94 BB630 (clone HP-3D9), CD127 BV650 (clone hIL-7R-M21), CD138 BB700 (clone MI15), CD150 BUV395 (clone A12), CD195 (CCR5) BB660 (clone 3A9), CD215 BV605 (clone JM7A4), T-bet BV786 (clone 04-46), RORγT BV421 (clone Q21-559), GzmB PE (clone GB11), GzmB Alexa700 (clone GB11) (all from BD Biosciences^®^), and Runx3-eFluor660 (clone R3-5G4) (eBioscience™). After incubation with specific antibodies against relevant surface molecules, PBMCs were fixed in BD Cytofix/CytoPerm solution for 30 min, washed with BD Perm/Wash buffer (BD Bioscience, San Diego, CA, USA), and then incubated overnight with intracellular markers. The acquisition was performed in FACSVerse^®^ and FACSymphony^®^ flow cytometers (BD Biosciences^®^), and the analysis used the FlowJo^®^ software.

### Isolating B Cells and *In Vitro* Stimulation

After the isolation from PBMCs using the EasySep^®^ Human B Cell Enrichment Kit with EasySep^®^ magnet, 2 × 10^4^ B cells were stimulated for 16 h in culture, with CPG-ODN (2.5 μl/ml) and human recombinant IL-21 (50 ng/ml) according to the literature (25, 26).

### Quantitative PCR

mRNA from isolated B cells was extracted using the RNeasy micro kit (QIAGEN) and reverse transcribed to cDNA. We used SYBR^®^ Green manufacturer’s instructions (BioRad, USA) to assess the expression of *GzmB* [*Forward* (F): CCATCCATCCAAGCCTATAATCCTA, *Reverse* (R): CCTGCACTGTCATCTTCACCT], *PRF1* (*F*: TGGAGTGCCGCTTCTACAGTT, *R*: GTGGGTGCCGTAGTTGGAGAT), and Runx3 (*F*: GAGTTTCACCCTGACCATCACTGTG, *R*: GCCCATCACTGGTCTTGAAGGTTGT). Data were normalized using a housekeeping gene *HPRT* (*F*: GACCAGTCAACAGGGGACAT, *R*: AACCTTCGTGGGGTCCTTTTC).

### Cytometric Bead Array

A total of 50 µl of isolated and stimulated B-cell supernatants and solutions for calibration curve construction were incubated with beads containing mAbs to GzmB. After incubation for 2 h, revealing antibody conjugated to the fluorochrome PE was added. The acquisition was performed in FACSCanto (BD Bioscience^®^) flow cytometer, and the analysis used the FCAP Array software (BD Bioscience^®^).

### Statistical Analyses

The statistical significance of the results was determined using a nonparametric analysis of variance (Kruskal–Wallis test) and a Mann–Whitney test (U-test). Dunn’s multiple comparison test was used as *post-hoc* of Kruskal–Wallis. The ROUT (Q = 1%)’ test was used to determine the presence of outlier values. *p* < 0.05 values were considered statistically significant.

## Results

### Granzyme B Expression in CD8^+^ T Lymphocytes From Relapsing-Remitting Multiple Sclerosis Patients

**Figure 1:**
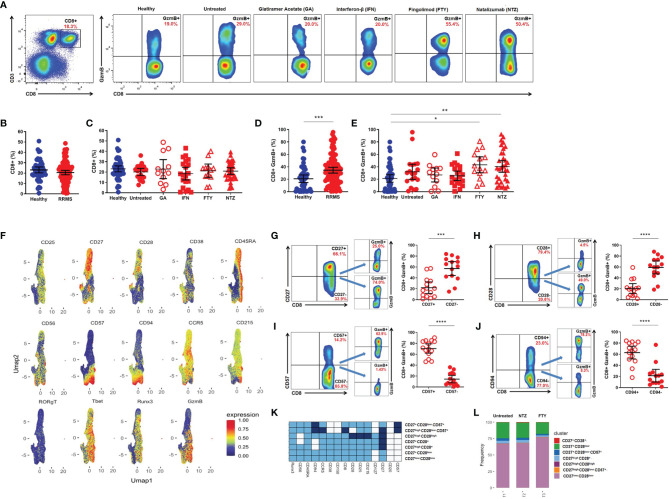
Cytotoxic CD8^+^ T lymphocytes in relapsing-remitting multiple sclerosis (RRMS) patients. **(A)** Gate strategy for total CD8^+^ and CD8^+^GzmB^+^ T lymphocytes from healthy donors, untreated RRMS, and treated [Glatiramer Acetate (GA), Interferon-β (IFN), Fingolimod (FTY), and Natalizumab (NTZ)] RRMS patients. **(B)** Proportion (%) of total CD8^+^ T lymphocytes in healthy donors (blue) and RRMS patients (red). **(C)** Proportion (%) of CD8^+^ T lymphocytes in healthy donors (blue), untreated RRMS (red), and treated RRMS patients (GA, IFN, FTY, NTZ) (red). **(D)** Proportion (%) of circulating CD8^+^GzmB^+^ T lymphocytes in healthy donors (blue) and RRMS patients (red). **(E)** Proportion (%) of circulating CD8^+^GzmB^+^ T lymphocytes in healthy donors (blue), untreated RRMS (red), and treated RRMS patients (GA, IFN, FTY, NTZ) (red). Bars represent mean values. Each column represents mean (95% CI). *p < 0.05; **p < 0.01; ***p < 0.001; ****p < 0.0001. **(F)** Uniform Manifold Approximation and Projection (UMAP) gated in CD3^+^CD8^+^ T lymphocytes from RRMS patients with different conditions non-identified and based on the Arcsinh-transformed expression of the markers. Gate strategy and proportion (%) of granzyme B (GzmB) derived from circulating **(G)** CD8^+^CD27^+^ vs. CD8^+^CD27^-^, **(H)** CD8^+^CD28^+^ vs. CD8^+^CD28^-^, **(I)** CD8^+^CD57^+^ vs. CD8^+^CD57^-^, **(J)** CD8^+^CD94^+^ vs. CD8^+^CD94^-^ T lymphocytes in RRMS patients (red). Bars represent mean values. Each column represents mean (95% CI). *p < 0.05; **p < 0.01; ***p < 0.001; ****p < 0.0001. **(K)** Heatmap of the expression of the markers in subpopulations manually identified in CD3^+^CD8^+^ T lymphocytes from RRMS patients. **(L)** Barplot representing the frequency of each subpopulation in CD3^+^CD8^+^ T lymphocytes.

Flow cytometry analysis of PBMCs (**Figure 1A**) showed no differences in the percentage of circulating CD8^+^ T lymphocytes from RRMS patients when compared to healthy donors. Subgroups from untreated RRMS or treated patients (GA, IFN, FTY, and NTZ) also showed no differences in comparison with healthy volunteers (**Figures 1B, C** **)**. However, an increased percentage of CD8^+^GzmB^+^ was found in the RRMS group vs. healthy donors (34.5 vs. 20.8, mean; 95% CI) (*p* < 0.0003) (**Figure 1D**). The expression of GzmB was also significantly higher in CD8^+^ T lymphocytes from patients treated with FTY (43.2, mean; 95% CI) (*p* = 0.0163) and NTZ (40.5, mean; 95% CI) (*p* = 0.0048) vs. healthy donors, but not in treated RRMS patients during first-line immunomodulatory therapies, GA and IFN (26.8 and 25.5, means; 95% CI) nor in untreated RRMS patients (31.9, mean; 95% CI) vs. healthy donors, respectively (**Figure 1E**). We then performed Uniform Manifold Approximation and Projection (UMAP) analyses in CD3^+^CD8^+^ T lymphocytes from untreated RRMS patients and treated RRMS patients during FTY or NTZ therapies. Various surface [CD25, CD27, CD28, CD38, CD45RA, CD56, CD57, CD94, CD127, CD150, CD195 (CCR5), CD215] and intracellular (RORγT, T-bet, Runx3) markers were used, aiming to concomitantly identify expression with GzmB. Using this strategy, we found senescent-associated markers such as CD28^-^ and CD57^+^, and more broadly CD27^-^ and CD94^+^, associated with GzmB expression in CD8^+^ T subsets from MS patients (**Figure 1F**). Upon confirming this, we assessed increased expression of GzmB in CD27^-^ vs. CD27^+^ (57.0 vs. 22.0, mean; 95% CI) (*p* = 0.0003) (**Figure 1G**), CD28^-^ vs. CD28^+^ (59.1 vs. 20.4, mean; 95% CI) (*p* < 0.0001) (**Figure 1H**), CD57^+^ vs. CD57^-^ (70.4 vs. 14.4, mean; 95% CI) (*p* < 0.0001) (**Figure 1I**), and CD94^+^ vs. CD94^-^ (62.9 vs. 21.3, mean; 95% CI) (*p* < 0.0001) (**Figure 1J**) markers of CD8^+^ T lymphocytes from RRMS patients. Moreover, heatmap analyses showed that CD27^low^CD28^low^ and CD27^+^CD28^low^ compose almost the totality of CD8^+^ T subsets from the investigated RRMS patients. Similar frequencies of these subsets were found in untreated RRMS patients and also in treated RRMS patients during FTY and NTZ (**Figures 1K, L** **)**.

### Granzyme B Expression in CD19^+^ B Cells From Relapsing-Remitting Multiple Sclerosis Patients

**Figure 2:**
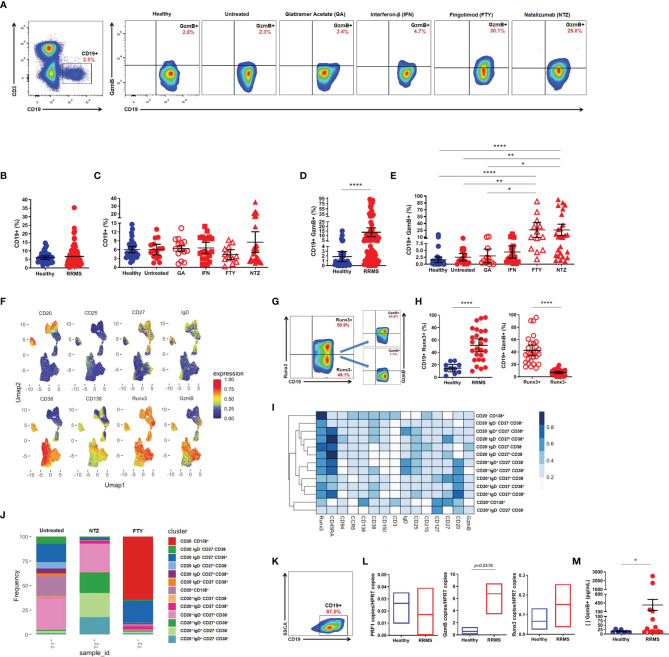
Cytotoxic CD19^+^ B cells in relapsing-remitting multiple sclerosis (RRMS) patients. **(A)** Gate strategy for total CD19^+^ and CD19^+^GzmB^+^ B cells from healthy donors, untreated RRMS, and treated [Glatiramer Acetate (GA), Interferon-β (IFN), Fingolimod (FTY), and Natalizumab (NTZ)] RRMS patients. **(B)** Proportion (%) of total CD19^+^ B cells in healthy donors (blue) and RRMS patients (red). **(C)** Proportion (%) of total CD19^+^ B cells in healthy donors (blue), untreated RRMS (red), and treated RRMS patients (GA, IFN, FTY, NTZ) (red). **(D)** Proportion (%) of circulating CD19^+^GzmB^+^ B cells in healthy donors (blue) and RRMS patients (red). **(E)** Proportion (%) of circulating CD19^+^GzmB^+^ B cells in healthy donors (blue), untreated RRMS (red), and treated RRMS patients (GA, IFN, FTY, NTZ) (red). Bars represent mean values. Each column represents mean (95% CI). *p < 0.05; **p < 0.01; ****p < 0.0001. **(F)** Uniform Manifold Approximation and Projection (UMAP) gated in CD3^-^CD19^+^ B cells from RRMS patients with different conditions non-identified and based on the Arcsinh-transformed expression of the markers. **(G)** Gate strategy for granzyme B (GzmB)-derived CD19^+^Runx3^+^ B cells. **(H)** Proportion (%) of GzmB-derived from circulating CD19^+^Runx3^+^ vs. CD19^+^Runx3^-^ B cells in healthy donors (blue) and RRMS patients (red). **(I)** Proportion (%) of GzmB derived from circulating CD19^+^Runx3^+^ vs. CD19^+^Runx3^-^ in RRMS patients (red). Each column represents mean (95% CI). *p < 0.05; **p < 0.01; ****p < 0.0001. **(I)** Heatmap of the expression of the markers in subpopulations manually identified in CD3^-^CD19^+^ B cells from RRMS patients. **(J)** Barplot representing the frequency of each subpopulation in CD3^-^CD19^+^ B cells. **(K)** Gate strategy for isolated CD19^+^ B cells. **(L)** Quantitative PCR for *PRF1*, *GzmB*, and *Runx3* in isolated B cells from healthy donors (blue) and RRMS patients (red). **(M)** Concentration (pg/ml) of GzmB in supernatants of stimulated CD19^+^ B cells from healthy donors (blue) and RRMS patients (red). Each column represents mean (SEM). *p < 0.05; **p < 0.01; ***p < 0.001; ****p < 0.0001.

Flow cytometry analysis (**Figure 2A**) did not reveal differences in the percentage of total circulating CD19^+^ B cells between RRMS patients and healthy donors (**Figure 2B**), nor among RRMS subgroups, despite the tendency of diminished circulating CD19^+^ B cells in FTY-treated patients (**Figure 2C**). However, an increased percentage of circulating CD19^+^GzmB^+^ B cells was found in RRMS patients vs. healthy donors (13.6 vs. 1.8, mean; 95% CI) (*p* < 0.0001) (**Figure 2D**). The expression of GzmB was also significantly higher in CD19^+^ B cells from patients treated with FTY when compared to GA (25.7 vs. 2.9, mean; 95% CI) (*p* = 0.0124), untreated patients (2.5, mean; 95% CI) (*p* = 0.0059), and healthy donors (1.8, mean; 95% CI) (*p* < 0.0001). Similarly, CD19^+^GzmB^+^ B cells were significantly higher in NTZ-treated patients (25.8, mean; 95% CI) concerning the first-line immunomodulatory therapy GA (*p* = 0.0109), untreated patients (*p* = 0.0037), and healthy donors (*p* < 0.0001) (**Figure 2E**). Statistical differences were not observed in FTY and NTZ subgroups when compared to IFN-treated patients (4.4, mean; 95% CI). Resembling the strategy for CD8^+^ T lymphocytes, we performed UMAP analyses in CD3^-^CD19^+^ B cells from untreated RRMS patients and treated RRMS patients during FTY or NTZ therapies. B cell-associated surface markers (CD20, CD25, CD27, CD38, CD138, IgD), as well as intracellular Runx3, were used, aiming to identify B subsets with the ability to express GzmB. Thus, we found that main GzmB-expressing B subsets lack the expression of CD20 marker but strongly correspond to CD38^+^ activation marker and CD138^+^ plasma cells (**Figure 2F**). We also notice a strong expression of Runx3 in B cells that concomitantly express GzmB. Upon confirming this, we assessed increased circulating CD19^+^Runx3^+^ in RRMS patients when compared to healthy donors (51.4 vs. 14.8, mean; 95% CI) (*p* < 0.0001). Moreover, we observed increased circulating GzmB-derived CD19^+^Runx3^+^ when compared to CD19^+^Runx3^-^ B cells from RRMS patients (42.4.8 vs. 6.9, mean; 95% CI) (*p* < 0.0001) (**Figures 2G, H**). Furthermore, including the previously mentioned markers, we were able to define distinct subsets of B cells in untreated and also in treated (FTY or NTZ) RRMS patients. Untreated RRMS patients mainly seem to exhibit CD20^+^ B subsets suggestive of antigen-activated switched memory phenotype (CD20^+^IgD^-^CD27^+^CD38^-^), non-classical plasma cells (CD20^+^CD138^+^), and CD20^-^ subsets also with atypical memory features (CD20^-^IgD^-^CD27^-^CD38^+/-^). Almost total of B subsets from NTZ-treated patients were CD20^+^ in which approximately half of them exhibited naive phenotype (CD20^+^IgD^+^CD27^-^CD38^+/-^) followed by memory subsets (CD20^+^IgD^-^CD27^-^CD38^+/-^). Finally, FTY-treated patients exhibited almost all of the B subsets lacking CD20 expression, suggesting well-known defined plasma cells (CD20^-^CD138^+^) and a few suggestive of early plasmablasts or memory cells (CD20^-^IgD^-^CD27^-^CD38^+^) (**Figures 2I, J** **)**.

### Release of Granzyme B by CD19^+^ B Cells Isolated From Relapsing-Remitting Multiple Sclerosis Patients

We sorted out CD19^+^ B cells to evaluate the *in vitro* cytotoxic activity (**Figure 2K**). After ODN-CPG and IL-21 stimulation, no differences regarding Perforin (*PRF1*), *GzmB*, or *Runx3* mRNA expression were found between isolated B cells from RRMS patients and healthy donors (**Figure 2L**). However, supernatants of purified CD19^+^ B cells from RRMS patients presented significantly higher amounts of GzmB in comparison with CD19^+^ B cells from healthy individuals (368.9 vs. 15.1, mean; SEM) (*p* = 0.0145) (**Figure 2M**).

## Discussion

Herein, we demonstrated that CD19^+^ B cells from RRMS patients share the ability to express serine-protease GzmB, similarly resembling classical CD8^+^ T lymphocytes.

Regarding T lymphocytes, we show here that RRMS patients exhibit an increased percentage of circulating CD8^+^GzmB^+^ T lymphocytes when compared to healthy volunteers. Moreover, treated RRMS patients, particularly FTY and NTZ subgroups, showed higher CD8^+^GzmB^+^ T lymphocytes than healthy subjects.

Enhanced cytotoxic behavior derived from T lymphocytes has been suggested as a mechanism for controlling latent Epstein–Barr virus (EBV) infection and preventing viral replication during MS (27). However, sustained cytotoxic CD8^+^ T cell activity would also be implicated in CNS lesions during disease. Indeed, infiltration of CD8^+^GzmB^+^ T lymphocytes that respond against EBV-infected B cells/plasma cells was recently found in the CNS lesions from two MS patients who died after suffering fulminant relapses following NTZ withdrawal (14, 28).

In addition, Cencioni et al. (4) showed that cytotoxic CD57^+^ T subsets occur in inflamed meninges from progressive MS patients and are negatively correlated with disease progression/age of death. Interestingly, higher expression of programmed death-1 (PD-1) in circulating CD8^+^CD57^+^ T lymphocytes correlates with disease stability. *In vitro* blockade of PD-1 enhanced the release of IFN-γ, Perforin, and GzmB by these terminally differentiated cytotoxic T subsets from MS patients (4).

According to previous reports (4, 29), RRMS patients in our cohort showed increased expression of GzmB in CD8^+^ T lymphocytes markedly associated with senescent T phenotype exhibiting CD27^-^, CD28^-^, CD57^+^, and CD94^+^ markers.

Indeed, diverse evidence suggests that cytotoxic subsets including those exhibiting senescent CD28^-^ and CD57^+^ markers restrain the migration ability into inflamed tissues in response to chemokines and also to express and release GzmB and other pro-inflammatory cytokines supporting tissue damage in diverse conditions (30).

Considering this, in the last few years, these subsets seemed to have emerged as candidates for predicting disease worsening in several diseases. The prognostic value of CD4^+^CD28^-^ T subset during MS was recently suggested for progressive disease (31). However, there is still a lack of studies investigating the potential of cytotoxic behavior in subsets other than T lymphocytes and its possible implications regarding different MS clinical courses/treatments.

On the other hand, the role of B cells during MS has been more deeply investigated in the context of antibody-independent functions. For instance, IL-21, which is known to promote B cell differentiation to memory and plasma cells in the presence of both BCR or Toll-like receptor (TLR) signaling and CD40L co-stimulation, may also promote GzmB-secreting B cells in the absence of CD40L co-stimulation (26, 32, 33).

Thus, considering that B cells may differentiate into GzmB-producing cells upon insufficient T cell help, herein, we have provided evidence that this phenomenon may occur during MS. Similar to CD8^+^ T lymphocytes, we found no differences in total circulating CD19^+^ B cells. However, our results show an increased percentage of circulating CD19^+^GzmB^+^ B cells in RRMS patients vs. healthy. Treated RRMS subgroup patients showed higher amounts of CD19^+^GzmB^+^ B cells during FTY and NTZ when compared to patients during first-line immunomodulatory therapy (GA), untreated RRMS patients, and healthy donors. We were able to assess which B-cell markers in CD3^-^CD19^+^ subsets were associated with the cytotoxic phenotype using flow cytometry high-dimensional analyses high-dimensional analyses. Accordingly, with previous literature, we observed that not CD20^+^ but CD38^+^ activated and CD138^+^ plasma cells seem to identify GzmB-expressing phenotype in B subsets (26, 34, 35). Moreover, Runx3, a master regulator associated with cytotoxic behavior in CD8^+^ T lymphocytes (8, 9) positively correlated with the GzmB-expressing phenotype.

As previously suggested by De Andrés et al. (36), these results reinforce a possible antibody-independent pathophysiological mechanism derived from B-cell subsets with the ability to express GzmB during MS. Beyond this, and considering the clinical efficacy of both FTY and NTZ, we may hypothesize that cytotoxicity may represent or even coexist with other tolerogenic functions in B subsets. Resembling our MS cohort, similar percentages of circulating CD19^+^GzmB^+^ B cells, in the absence of IL-10 coexpression, were described during HIV infection (37). Also, regulatory activity of GzmB-derived circulating CD19^+^ B cells was suggested due to degradation of TCR-ζ–chain that promotes a significant decrease in T-cell proliferation (32, 37, 38). As our results suggest, by now, it seems that GzmB expression is mainly derived from CD20^-^ B subsets with CD38^+^ and CD138^+^ markers. Indeed, beyond several changes regarding the total percentage of CD19^+^ B cells comprehending naive and memory phenotypes, as well as regulatory B subsets, increased circulating plasma cells were already described during highly effective MS treatments (39). Interestingly, reduced tumor necrosis factor (TNF)-α and enhanced interleukin (IL)-10 expression by B subsets were also reported during FTY. Yet, these regulatory IL-10-expressing B cells seem to be increased in the CSF from FTY-treated patients (24, 40).

It is noteworthy that despite CD20^+^ B cells being found in CNS lesions from different stages of the disease, many authors have proposed that B cells would take a later role in MS pathophysiology, since, in 2004, CD20^+^ B cells, CD138^+^ plasma cells, and follicular dendritic cells were described in tertiary lymphoid organs in inflamed meninges from progressive MS patients (5, 27).

Further investigation in progressive MS courses may identify whether or not GzmB-derived B cells occur during chronic disease pathogenesis. So far, cytotoxicity derived from B cells was shown to cause damage in oligodendrocytes and neurons (41, 42), eventually sustaining a silent and continuous CNS-restricted inflammatory process. Supporting this, anti-CD20 mAbs seem to be effective for managing progressive MS mainly during early disease course (24, 40, 43) and have also been suggested for mitigating the increased risk of relapses in RRMS patients after NTZ washout (44).

Thus, since anti-CD20 mAbs mainly deplete naive and memory B cells, preserving antibody-secreting (CD138+) plasma cells, cytotoxic behavior derived from CD20^-^ B subsets would be preserved during these treatments. Further investigations of cytotoxic behavior in CD19^+^ may address, for instance, eventual important mechanisms associated with the clinical efficacy of emerging anti-CD19 mAbs and oral drugs targeting Bruton’s tyrosine kinase (BTK) for MS patients (45).

### Conclusions

Our findings collectively support that beyond classical CD8^+^ T subsets, CD19^+^ B cells may be an alternative source of lytic factors such as GzmB in the context of antibody-independent functions during MS.

### Limitations

The size of cohort and the cross-sectional nature of our study did not allow us to understand the clinical relevance of our findings better. Although we were able to establish a strong correlation between Runx3 and GzmB expression, *in vitro* generation of cytotoxic B cells will be necessary to clarify the role of Runx3 expression in this subset.

## Data Availability Statement

The raw data supporting the conclusions of this article will be made available by the authors without undue reservation.

## Ethics Statement

The studies involving human participants were reviewed and approved by the University of Campinas Committee for Ethical Research (CAAE: 53022516.3.0000.5404). The patients/participants provided their written informed consent to participate in this study.

## Author Contributions

VB, RQ, and ASM performed most of the experiments. CS, AD, RC, and CB diagnosed, treated, and selected MS patients as well as recruited all healthy donors. VB, AMM, ASM, MA, and BF designed and performed flow cytometry. VB, AMM, and AF analyzed flow cytometry data. AL and IS performed CBA experiments. AF and LS designed the experimental work. AF coordinated the study. VB, AMM, AF, and LS wrote the article with inputs from co-authors. All authors contributed to the article and approved the submitted version.

## Funding

This work was supported by grants from Sao Paulo Research Foundation (FAPESP) (#2014/26431-0, 2015/22052-8, #2017/21363-5, #2019/06372-3, #2019/16116-4). This study was also partly financed in part by the Coordenação de Aperfeiçoamento de Pessoal de Nível Superior-Brasil (CAPES)-Finance Code 001 (2015/22052-8).

## Conflict of Interest

LS received a research grant from Biogen and a consultation honorarium from Biogen and Roche.

The remaining authors declare that the research was conducted in the absence of any commercial or financial relationships that could be construed as a potential conflict of interest.

## Publisher’s Note

All claims expressed in this article are solely those of the authors and do not necessarily represent those of their affiliated organizations, or those of the publisher, the editors and the reviewers. Any product that may be evaluated in this article, or claim that may be made by its manufacturer, is not guaranteed or endorsed by the publisher.

## References

[1] Neumann H Medana IM Bauer J Lassmann H. Cytotoxic T Lymphocytes in Autoimmune and Degenerative CNS Diseases. Trends Neurosci (2002) 25:313–9. doi: 10.1016/S0166-2236(02)02154-9 12086750

[2] Skulina C Schmidt S Dornmair K Babbe H Roers A Rajewsky K. Multiple Sclerosis: Brain-Infiltrating CD8+ T Cells Persist as Clonal Expansions in the Cerebrospinal Fluid and Blood. Proc Comput Sci (2004) 101:2428–33. doi: 10.1073/PNAS.0308689100 PMC356967 14983026

[3] Ifergan I Kebir H Alvarez JI Marceau G Bernard M Bourbonniere L. Central Nervous System Recruitment of Effector Memory CD8+ T Lymphocytes During Neuroinflammation is Dependent on 4 Integrin. Brain (2011) 134:3560–77. doi: 10.1093/brain/awr268 PMC7110084 22058139

[4] Cencioni MT Magliozzi R Nicholas R Ali R Malik O Reynolds R. Programmed Death 1 is Highly Expressed on CD8+ CD57+ T Cells in Patients With Stable Multiple Sclerosis and Inhibits Their Cytotoxic Response to Epstein–Barr Virus. Immunology (2017) 152:660–76. doi: 10.1111/imm.12808 PMC5680058 28767147

[5] Lassmann H. Pathogenic Mechanisms Associated With Different Clinical Courses of Multiple Sclerosis. Front Immunol (2019) 9:3116. doi: 10.3389/fimmu.2018.03116 30687321 PMC6335289

[6] Merelli E Sola P Faglioni P Giordani S Mussini D Montagnani G. Natural Killer Cells and Lymphocyte Subsets in Active MS and Acute Inflammation of the CNS. Acta Neurol Scand (1991) 84:127–31. doi: 10.1111/j.1600-0404.1991.tb04920.x 1835239

[7] Chiang SCC Theorell J Entesarian M Meeths M Mastafa M Al-Herz W. Comparison of Primary Human Cytotoxic T-Cell and Natural Killer Cell Responses Reveal Similar Molecular Requirements for Lytic Granule Exocytosis But Differences in Cytokine Production. Blood (2013) 121:1345–56. doi: 10.1182/blood-2012-07-442558 23287865

[8] Cruz-Guilloty F Pipkin ME Djuretic IM Levanon D Lotem J Lichtenheld MG. Runx3 and T-Box Proteins Cooperate to Establish the Transcriptional Program of Effector CTLs. J Exp Med (2009) 206:51–9. doi: 10.1084/jem.20081242 PMC2626671 19139168

[9] Behr FM Chuwonpad A Stark R van Gisbergen KPJM. Armed and Ready: Transcriptional Regulation of Tissue-Resident Memory CD8 T Cells. Front Immunol (2018) 9:1770. doi: 10.3389/fimmu.2018.01770 30131803 PMC6090154

[10] Himmelstein DS Baranzini SE. Heterogeneous Network Edge Prediction : A Data Integration Approach to Prioritize Disease-Associated Genes. PLoS Comput Biol (2015) 11(7):e1004259. doi: 10.1371/journal.pcbi.1004259 26158728 PMC4497619

[11] Haile Y Carmine-Simmen K Olechowski C Kerr B Bleackley RC Giuliani F. Granzyme B-Inhibitor Serpina3n Induces Neuroprotection *In Vitro* and *In Vivo* . J Neuroinflamm (2015) 12:1–10. doi: 10.1186/s12974-015-0376-7 PMC4558826 26337722

[12] Malmeström C Lycke J Haghighi S Andersen O Carlsson L Wadenvik H. Relapses in Multiple Sclerosis are Associated With Increased CD8+ T-Cell Mediated Cytotoxicity in CSF. J Neuroimmunol (2008) 196:159–65. doi: 10.1016/j.jneuroim.2008.03.001 18396337

[13] Fujii C Kondo T Ochi H Okada Y Hashi Y Adachi T. Altered T Cell Phenotypes Associated With Clinical Relapse of Multiple Sclerosis Patients Receiving Fingolimod Therapy. Sci Rep (2016) 6:35314. doi: 10.1038/srep35314 27752051 PMC5082790

[14] Serafini B Scorsi E Rosicarelli B Rigau V Thouvenot E Aloisi F. Massive Intracerebral Epstein-Barr Virus Reactivation in Lethal Multiple Sclerosis Relapse After Natalizumab Withdrawal. J Neuroimmunol (2017) 307:14–7. doi: 10.1016/j.jneuroim.2017.03.013 28495131

[15] Larochelle C Metz I Lécuyer M Terouz S Roger M Arbour N. Immunological and Pathological Characterization of Fatal Rebound MS Activity Following Natalizumab Withdrawal. Mult Scler J (2017) 23:72–81. doi: 10.1177/1352458516641775 27037182

[16] Lee PR Johnson TP Gnanapavan S Giovannoni G Wang T Steiner JP. Protease-Activated Receptor-1 Activation by Granzyme B Causes Neurotoxicity That is Augmented by Interleukin-1β. J Neuroinflamm (2017) 14:1–18. doi: 10.1186/s12974-017-0901-y PMC5488439 28655310

[17] Sauer BM Schmalstieg WF Howe CL. Axons are Injured by Antigen-Specific CD8+ T Cells Through a MHC Class I- and Granzyme B-Dependent Mechanism. Neurobiol Dis (2013) 59:194–205. doi: 10.1016/j.nbd.2013.07.010 23899663 PMC3788647

[18] Salou M Nicol B Garcia A Laplaud D-A. Involvement of CD8+ T Cells in Multiple Sclerosis. Front Immunol (2015) 6:604. doi: 10.3389/fimmu.2015.00604 26635816 PMC4659893

[19] Sellebjerg F Bornsen L Khademi M Krakauer M Olsson T Frederiksen JL. Increased Cerebrospinal Fluid Concentrations of the Chemokine CXCL13 in Active MS. Neurology (2009) 73:2003–10. doi: 10.1212/WNL.0b013e3181c5b457 19996075

[20] Krumbholz M Derfuss T Hohlfeld R Meinl E. B Cells and Antibodies in Multiple Sclerosis Pathogenesis and Therapy. Nat Rev Neurol (2012) 8:613–23. doi: 10.1038/nrneurol.2012.203 23045237

[21] Ferraro D Simone AM Bedin R Galli V Vitetta F Federzoni L. Cerebrospinal Fluid Oligoclonal IgM Bands Predict Early Conversion to Clinically Definite Multiple Sclerosis in Patients With Clinically Isolated Syndrome. J Neuroimmunol (2013) 257:76–81. doi: 10.1016/j.jneuroim.2013.01.011 23434160

[22] Krumbholz M Meinl E. B Cells in MS and NMO: Pathogenesis and Therapy. Semin Immunopathol (2014) 36:339–50. doi: 10.1007/s00281-014-0424-x 24832354

[23] Holloman JP Axtell RC Monson NL Wu GF. The Role of B Cells in Primary Progressive Multiple Sclerosis. Front Neurol (2021) 12:680581. doi: 10.3389/fneur.2021.680581 34163430 PMC8215437

[24] Li R Rezk A Healy LM Muirhead G Prat A Gommerman JL. Cytokine-Defined B Cell Responses as Therapeutic Targets in Multiple Sclerosis. Front Immunol (2016) 6:626. doi: 10.3389/fimmu.2015.00626 26779181 PMC4705194

[25] Jahrsdörfer B Vollmer A Blackwell SE Maier J Sontheimer K Beyer T. Granzyme B Produced by Human Plasmacytoid Dendritic Cells Suppresses T-Cell Expansion. Blood (2010) 115:1156–65. doi: 10.1182/blood-2009-07-235382 PMC2920226 19965634

[26] Cupi ML Sarra M Marafini I Monteleone I Franze E Ortenzi A. Plasma Cells in the Mucosa of Patients With Inflammatory Bowel Disease Produce Granzyme B and Possess Cytotoxic Activities. J Immunol (2014) 192:6083–91. doi: 10.4049/jimmunol.1302238 24835396

[27] Serafini B Rosicarelli B Magliozzi R Stigliano E Aloisi F. Detection of Ectopic B-Cell Follicles With Germinal Centers in the Meninges of Patients With Secondary Progressive Multiple Sclerosis. Brain Pathol (2004) 14:164–74. doi: 10.1111/j.1750-3639.2004.tb00049.x PMC8095922 15193029

[28] Serafini B Zandee S Rosicarelli B Scorsi E Veroni C Larochelle C. Epstein-Barr Virus-Associated Immune Reconstitution Inflammatory Syndrome as Possible Cause of Fulminant Multiple Sclerosis Relapse After Natalizumab Interruption. J Neuroimmunol (2018) 319:9–12. doi: 10.1016/j.jneuroim.2018.03.011 29685294

[29] Broux B Mizee MR Vanheusden M van der Pol S van Horssen J Van Wijmeersch B. IL-15 Amplifies the Pathogenic Properties of CD4 + CD28 – T Cells in Multiple Sclerosis. J Immunol (2015) 194:2099–109. doi: 10.4049/jimmunol.1401547 25617471

[30] Darrah E Rosen A. Granzyme B Cleavage of Autoantigens in Autoimmunity. Cell Death Differ (2010) 17:624–32. doi: 10.1038/cdd.2009.197 PMC3136751 20075942

[31] Peeters LM Vanheusden M Somers V van Wijmeersch B Stinissen P Broux B. Cytotoxic CD4+ T Cells Drive Multiple Sclerosis Progression. Front Immunol (2017) 8:1160. doi: 10.3389/fimmu.2017.01160 28979263 PMC5611397

[32] Hagn M Jahrsdörfer B. Why do Human B Cells Secrete Granzyme B? Insights Into a Novel B-Cell Differentiation Pathway. Oncoimmunology (2012) 1:1368–75. doi: 10.4161/onci.22354 PMC3518509 23243600

[33] Hagn M Sontheimer K Dahlke K Brueggemann S Kaltenmeier C Beyer T. Human B Cells Differentiate Into Granzyme B-Secreting Cytotoxic B Lymphocytes Upon Incomplete T-Cell Help. Immunol Cell Biol (2012) 90:457–67. doi: 10.1038/icb.2011.64 21808264

[34] Chesneau M Michel L Dugast E Chenouard A Baron D Pallier A. Tolerant Kidney Transplant Patients Produce B Cells With Regulatory Properties. J Am Soc Nephrol (2015) 26:2588–98. doi: 10.1681/ASN.2014040404 PMC4587683 25644114

[35] Chesneau M Le MH Danger R Le Bot S Nguyen T-V-H Bernard J. Efficient Expansion of Human Granzyme B–Expressing B Cells With Potent Regulatory Properties. J Immunol (2020) 205:2391–401. doi: 10.4049/jimmunol.2000335 32948686

[36] De Andrés C Tejera-Alhambra M Alonso B Valor L Teijeiro R Ramos-Medina R. New Regulatory CD19+CD25+B-Cell Subset in Clinically Isolated Syndrome and Multiple Sclerosis Relapse. Changes After Glucocorticoids. J Neuroimmunol (2014) 270:37–44. doi: 10.1016/j.jneuroim.2014.02.003 24662004

[37] Kaltenmeier C Gawanbacht A Beyer T Lindner S Trzaska T van der Merwe JA. CD4+ T Cell-Derived IL-21 and Deprivation of CD40 Signaling Favor the *In Vivo* Development of Granzyme B-Expressing Regulatory B Cells in HIV Patients. J Immunol (2015) 194:3768–77. doi: 10.4049/jimmunol.1402568 25780036

[38] Lindner S Dahlke K Sontheimer K Hagn M Kaltenmeier C Barth TFE. Interleukin 21-Induced Granzyme B-Expressing B Cells Infiltrate Tumors and Regulate T Cells. Cancer Res (2013) 73:2468–79. doi: 10.1158/0008-5472.CAN-12-3450 23384943

[39] Nakamura M Matsuoka T Chihara N Miyake S Sato W Araki M. Differential Effects of Fingolimod on B-Cell Populations in Multiple Sclerosis. Mult Scler J (2014) 20:1371–80. doi: 10.1177/1352458514523496 24526661

[40] Li R Patterson KR Bar-Or A. Reassessing B Cell Contributions in Multiple Sclerosis. Nat Immunol (2018) 19:696–707. doi: 10.1038/s41590-018-0135-x 29925992

[41] Lisak RP Benjamins JA Nedelkoska L Barger JL Ragheb S Fan B. Secretory Products of Multiple Sclerosis B Cells are Cytotoxic to Oligodendroglia. Vitro J Neuroimmunol (2012) 246:85–95. doi: 10.1016/j.jneuroim.2012.02.015 22458983

[42] Lisak RP Nedelkoska L Benjamins JA Schalk D Bealmear B Touil H. B Cells From Patients With Multiple Sclerosis Induce Cell Death *via* Apoptosis in Neurons *In Vitro* . J Neuroimmunol (2017) 309:88–99. doi: 10.1016/j.jneuroim.2017.05.004 28601295

[43] Filippi M Bar-Or A Piehl F Preziosa P Solari A Vukusic S. Multiple Sclerosis. Nat Rev Dis Prim (2018) 4:43. doi: 10.1038/s41572-018-0041-4 30410033

[44] Alping P Frisell T Novakova L Islam-Jakobsson P Salzer J Björck A. Rituximab Versus Fingolimod After Natalizumab in Multiple Sclerosis Patients. Ann Neurol (2016) 79:950–8. doi: 10.1002/ana.24651 27038238

[45] Sellebjerg F Blinkenberg M Sorensen PS. Anti-CD20 Monoclonal Antibodies for Relapsingand Progressive Multiple Sclerosis. CNS Drugs (2020) 34:269–80. doi: 10.1007/s40263-02000704-w 31994023

